# Programmable ROS modulation by nanomedicine for rheumatoid arthritis treatment

**DOI:** 10.1016/j.mtbio.2025.102699

**Published:** 2025-12-18

**Authors:** Guojun Pan, Yaxin Zhang, Di Liu, Yongbin Wang, Hengzhen Zhang, Haoen Pan, Qingfan Hou, Xiangrui Kong, Fujin Lv, Na Xiao, Renshuai Zhang

**Affiliations:** a School of Life Sciences, Shandong First Medical University & Shandong Academy of Medical Sciences, Jinan, 250117, PR China; b School of Basic Medical Sciences, Xinjiang Medical University, 830011, PR China; c College of Agronomy, Shandong Agricultural University, Tai'an, Shandong, 271018, China; d School of Pharmaceutical Sciences & Institute of Materia Medica, Shandong First Medical University & Shandong Academy of Medical Sciences, Jinan, 250117, PR China

**Keywords:** Rheumatoid arthritis, Reactive oxygen species, Nanomedicine, ROS-Responsive delivery, Multimodal therapy, Immuno-microenvironment modulation

## Abstract

Rheumatoid arthritis (RA) is a chronic autoimmune disease characterized by persistent synovial inflammation and progressive joint destruction. Extensive studies have demonstrated that reactive oxygen species (ROS) play a critical role in the pathogenesis and progression of RA. Imbalanced ROS not only aggravate synovial inflammation and cartilage degradation but also disrupt immune homeostasis. In recent years, nanotechnology-based strategies for regulating ROS have provided new insights into the precise treatment of RA. This review systematically summarizes ROS-centered nanotherapeutic systems applied in RA therapy, including ROS-scavenging nanosystems, ROS-responsive nanosystems, ROS-scavenging and responsive composite nanosystems, and ROS-augmenting nanosystems. Furthermore, the review highlights the advantages of ROS-modulating nanosystems in reshaping the immune microenvironment, restoring redox balance, and achieving combination therapy and theranostic integration, while also addressing challenges related to biosafety, controllability, and clinical translation. Overall, programmable ROS-modulating nanotherapeutic strategies offer new directions and theoretical foundations for the precise treatment of RA.

## Highlights

• Programmable ROS modulation framed as a core strategy for RA nanotherapy.
• Link ROS biology, inflammatory pathways and nanomaterial design principles.
• Summarize ROS-scavenging, ROS-responsive, hybrid and ROS-augmenting systems.
• Discuss translational challenges and design cues for clinical RA nanomedicine.

## Graphical abstract

**Figure:**
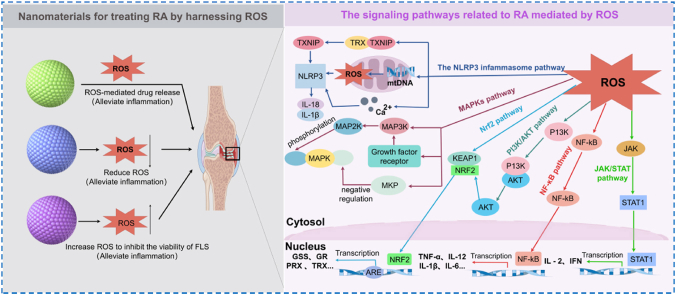

## 1 Introduction

**Fig. 1:**
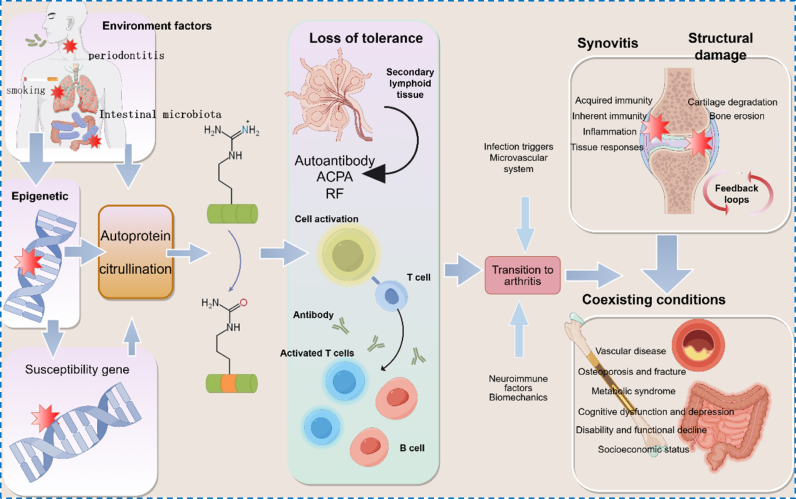
Multi-stage progression of rheumatoid arthritis (RA). ACPA, anti-citrullinated protein antibodies; RF, rheumatoid factor.

Rheumatoid arthritis (RA) is a common chronic systemic autoimmune disease characterized by persistent synovial inflammation, progressive cartilage and bone destruction, and significant extra-articular involvement [1,2]. Clinically, patients typically present with symmetrical polyarthritis of the small joints in the hands and feet, accompanied by joint pain, swelling, and morning stiffness, which can ultimately lead to disability and impaired quality of life [3,4]. Beyond articular damage, RA is associated with increased risks of cardiovascular and pulmonary diseases, osteoporosis, and other comorbidities, underscoring its substantial systemic and socioeconomic burden [[5], [6], [7], [8], [9]]. At the mechanistic level, RA arises from interactions between genetic susceptibility and environmental triggers such as cigarette smoking, which break immune tolerance and drive the production of autoantibodies, including anti-citrullinated protein antibodies (ACPA) and rheumatoid factor (RF) (Fig. 1) [[10], [11], [12]]. These maladaptive immune responses converge on the synovium, where persistent inflammatory cell infiltration, pannus formation, and osteoclast activation result in structural damage to cartilage and bone [[13], [14], [15], [16]].

Reactive oxygen species (ROS) occupy a pivotal position in the mechanisms underlying RA development [17]. The principal ROS species comprise superoxide (O_2_^−^), hydroxyl radical (·OH), hydrogen peroxide (H_2_O_2_) and singlet oxygen (^1^O_2_) [18]. Under normal physiological conditions, the production and clearance of ROS are maintained in a dynamic equilibrium. However, in RA patients, ROS levels remain elevated in the joint synovium and inflammatory microenvironments, resulting in oxidative stress [19]. Oxidative stress not only damages biomolecules, including DNA, lipids, and proteins, but also exacerbates local hypoxia, enhancing the activation of pro-inflammatory pathways, thus intensifying chronic inflammation [20,21]. Furthermore, ROS also function as signaling molecules that regulate immune cell activation, polarization, and the expression of inflammatory mediators. They modulate the functions of multiple immune populations, including T cells, B cells, and macrophages, thereby promoting the inflammatory and destructive processes associated with RA [22,23]. As a result, targeting ROS regulation has emerged as a promising therapeutic strategy in the management of RA [24].

Currently, pharmacotherapy is the cornerstone of RA management, encompassing nonsteroidal anti-inflammatory drugs, corticosteroids, disease-modifying antirheumatic drugs, and novel targeted therapies (such as JAK inhibitors, monoclonal antibodies, etc.) [25,26]. Although these therapies can effectively control symptoms and slow disease progression, many patients still experience incomplete responses or disease flares, and long-term systemic administration is often limited by adverse events, including hepatotoxicity, nephrotoxicity, and increased infection risk [[27], [28], [29], [30]]. Nanomedicine offers opportunities to overcome part of these limitations by improving drug solubility and stability, prolonging circulation time, and enabling site-directed and stimulus-responsive delivery within the inflamed joint microenvironment [[31], [32], [33], [34], [35], [36], [37], [38], [39]]. By enhancing intra-articular drug accumulation at lower doses and reducing off-target exposure, nanodrug systems provide innovative strategies for RA treatment and have therefore attracted intense research interest. Additionally, nanomaterials exhibit favorable biocompatibility, effectively reducing toxicity [[40], [41], [42]].

**Fig. 2:**
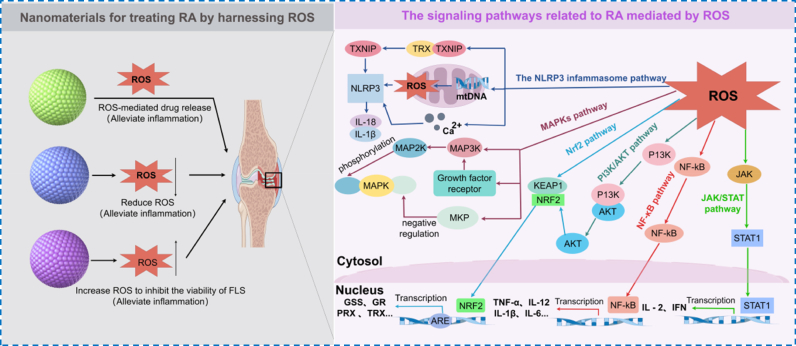
Classification of ROS-regulated nanomedicine systems and inflammatory signal pathways involving RA mediated by ROS.

Notably, in response to the microenvironment of RA joint lesions characterized by sustained high expression of ROS, ROS-regulated nanomedicine systems have emerged [43]. As illustrated in Fig. 2, nanomedicines can function as ROS scavengers to alleviate oxidative stress, or through ROS-responsive carriers, enable precise drug release in high-ROS environments, thus effectively intervening in inflammatory responses and tissue damage [43,44]. Moreover, studies have employed photodynamic therapy (PDT) and other approaches to locally elevate ROS levels, thereby inhibiting abnormal synovial cell proliferation and expanding the therapeutic applications of nanomedicine systems [45]. Nanomaterials intervene in the progression of RA through multiple mechanisms, with ROS acting as a central second messenger in regulating inflammation-related signaling pathways [46,47]. ROS can directly trigger the NOD-like receptor pyrin domain-containing 3 (NLRP3) inflammasome, thereby amplifying articular inflammation via release of oxidized mitochondrial DNA and calcium-dependent activation [48]. In parallel, ROS modulate the NF-κB and MAPK axes: oxidation of IKKβ lifts IκB-mediated restraint, enabling NF-κB nuclear translocation and transcription of pro-inflammatory mediators such as TNF-α and IL-6, while concomitant activation of MAPK signaling together accelerates cartilage degradation [49]. ROS also modulate cytoprotective signaling-by oxidizing Kelch-like ECH-associated protein 1, they release Nuclear factor erythroid 2-related factor 2 (Nrf2) to drive antioxidant gene expression and preserve redox balance, although chronic ROS elevation culminates in Nrf2 pathway fatigue and dysfunction. On the other hand, oxidative stress inhibits phosphatase and tensin homology (PTEN) phosphatase activity, activating the PI3K/AKT pathway, which enhances the abnormal proliferation of synovial cells by promoting NF-κB, while AKT further boosts Nrf2's transcriptional activity through phosphorylation, initiating a compensatory antioxidant mechanism [50]. Ultimately, a feedback loop emerges with ROS as the messenger: interleukin-1β (IL-1β) released by NLRP3 feedback activates NF-κB, while the PI3K/AKT and MAPK pathways synergistically drive synovial cell proliferation and bone erosion [51]. Meanwhile, Nrf2 pathway exhaustion and PTEN oxidative inactivation lead to the collapse of antioxidant defense mechanisms [52]. Based on these findings, nanomedicines can be designed to respond to ROS, directly scavenge ROS, or even increase ROS, thereby precisely modulating these signaling networks to alleviate inflammation and reduce tissue damage.

Recently, several comprehensive reviews have summarized nanomedicine-based strategies for RA [[53], [54], [55], [56], [57]]. For example, Han et al. summarize the present state of anti-RA nano-drug, focusing on providing an in-depth overview of nanomedicines that enhance the efficacy and safety of conventional anti-rheumatic drugs, while Qama et al. highlighted theranostic nanoplatforms that integrate imaging, targeted delivery, and multimodal therapy for RA management [53,54]. These reviews primarily categorize nanomedicines according to carrier type, targeting strategy, or therapeutic modality, and generally consider ROS as one of several pathological factors rather than the central organizing concept. In contrast, the present review is centered on the concept of programmable ROS modulation in RA. Here, we systematically classify ROS-oriented nanomedicine strategies into four major categories: (i) ROS-scavenging nanosystems that neutralize excessive ROS and restore redox homeostasis; (ii) ROS-responsive nanosystems that exploit elevated ROS as a trigger for on-demand drug release; (iii) hybrid ROS-responsive and ROS-scavenging composite nanosystems that couple stimulus-responsive delivery with intrinsic ROS-eliminating capacity; and (iv) ROS-augmenting nanosystems that locally amplify ROS to induce immunogenic cell death or synergize with photodynamic, photothermal, or sonodynamic therapy. By integrating ROS biology, inflammatory signaling pathways, and nanomaterial design principles, we aim to provide a mechanistically oriented framework that may better guide the rational design of next-generation ROS-modulating nanomedicines for RA.

## 2 Nanosystems for RA treatment based on ROS

### 2.1 ROS-scavenging nanosystems for RA treatment

**Fig. 3:**
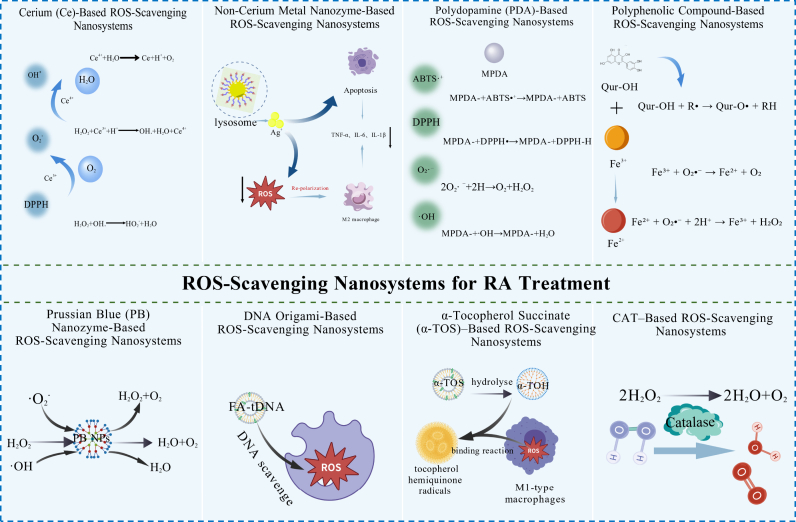
Schematic illustration of the ROS-scavenging mechanism of nanosystems in RA treatment.

In RA, joint tissues are heavily infiltrated by inflammatory leukocytes, particularly macrophages, neutrophils, and T cells [[58], [59], [60]]. Under inflammatory conditions, activation of these cells triggers a respiratory burst that generates high ROS levels. Excess ROS contributes to multiple pathogenic processes, including the secretion of pro-inflammatory cytokines, differentiation and activation of osteoclasts, and polarization of macrophages toward pro-inflammatory phenotypes [61]. Consequently, dysregulated ROS homeostasis is a central feature of RA pathobiology. Therapeutic strategies aimed at reducing excessive ROS, such as antioxidant therapies and ROS-scavenging agents, are therefore highly relevant for RA management [62,63]. Representative ROS-scavenging nanosystems are summarized in Fig. 3, and selected examples are discussed in detail in the following sections.

#### 2.1.1 Cerium-based ROS-scavenging nanosystems

Cerium ions (Ce^3+^/Ce^4+^) exhibit remarkable ROS scavenging capabilities due to their reversible valence state switching. In vivo, through Ce^3+^/Ce^4+^ redox cycling, cerium-oxide nanozymes exhibit superoxide-dismutase-like activity (catalyzing O_2_^−^ dismutation to H_2_O_2_ and O_2_) and catalase (CAT)-like activity (decomposing H_2_O_2_ into H_2_O and O_2_) [64]. In addition, Ce ions function as nanozyme mimics of key antioxidant enzymes, including superoxide dismutase (SOD) and CAT, effectively catalyzing the dismutation of O_2_^−^ into H_2_O_2_ and O_2_, and further decomposing H_2_O_2_ [[65], [66], [67], [68]]. Due to the reversible redox properties of Ce ions with variable valence states, Ce-based nanostructures can efficiently scavenge various ROS in the inflammatory environment of RA [69,70]. These platforms have demonstrated encouraging efficacy in preclinical models of RA.

**Fig. 4:**
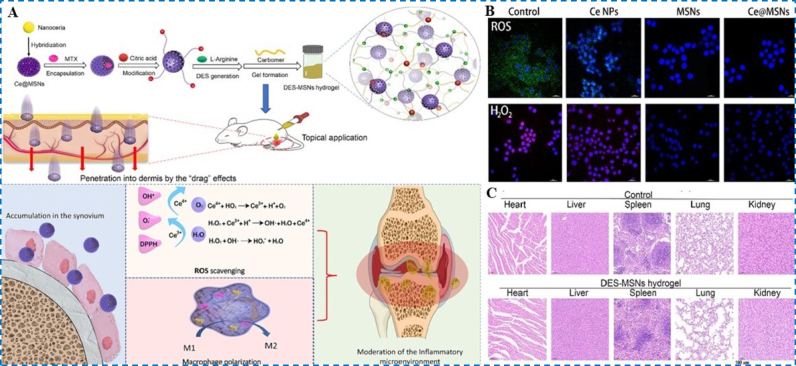
(A) Workflow for fabricating Ce@MSNs and overview of the mechanistic pathways relevant to RA treatment. (B) Qualitative analysis of ROS- and H_2_O_2_-scavenging effects in macrophages. (C) H&E examination of the major organs. Reproduced with permission from Ref. [71]. Copyright Elsevier 2023.

Owing to their switchable Ce^3+^/Ce^4+^ states and broad enzyme-mimetic activities, cerium ions have garnered substantial interest for RA nanotherapy. Li et al. developed a novel hydrogel system (Ce@MSNs) by immobilizing cerium oxide (CeO_2_) nanoparticles onto silica nanoparticles and loading them with methotrexate (MTX) (Fig. 4A) [71]. In this system, MTX inhibits dihydrofolate reductase, disrupting folate (FA) metabolism and thereby suppressing immune cell proliferation and inflammatory responses to alleviate RA symptoms. In parallel, Ce-based nanoparticles eliminate ROS and bias macrophages toward an M2 state, contributing to the modulation of local inflammation. In vitro studies demonstrated that Ce@MSNs exhibit enhanced scavenging capabilities for oxygen radicals, ·OH, and DPPH radicals, significantly reducing ROS levels within macrophages (Fig. 4B). Permeation experiments confirmed that this system can penetrate rat skin and distribute uniformly to deeper layers. In mice with RA, the Ce@MSNs hydrogel reduced inflammatory cytokines to 53 % (TNF-α), 44 % (Caspase-1), 54 % (IL-6), and 58 % (IL-1β) relative to controls, underscoring its potent anti-inflammatory effects. Additionally, biosafety assessments confirmed that the hydrogel exhibits low toxicity (Fig. 4C).

Irina Kalashnikova et al. utilized albumin as a carrier to prolong the in vivo circulation time of nanoparticles and enhance their targeted delivery to inflammatory regions [64]. Cerium oxide can synergistically clear ROS through multi-enzyme action, regulate hypoxia, and induce M2 macrophage polarization, effectively alleviating joint inflammation and swelling. Indocyanine green (ICG) imaging further confirmed high accumulation at the inflammatory site. Jonghoon Kim et al. combined the oxygen-producing Fenton reaction of manganese ferrite with the ROS scavenging ability of CeO_2_ to achieve synergistic relief from hypoxia and oxidative stress, promoting the transformation of macrophages toward an anti-inflammatory phenotype [72]. Polyethylene glycol (PEG) modification enhanced in vivo stability and biocompatibility, demonstrating superior anti-inflammatory effects in animal experiments. Additionally, Fu et al. combined magnesium aluminium layered double hydroxide with CeO_2_ nanoparticles, leveraging the pH responsiveness and acid-neutralizing properties of magnesium aluminium layered double hydroxide to boost the system's antioxidant activity [73,74]. This significantly promoted M2 polarization in joints and improved pathological manifestations in RA mice. While these nanoscale systems exhibit innovative features in terms of material structure, delivery pathways, and synergistic mechanisms, they collectively validate the universal mechanism of alleviating RA through efficient ROS scavenging and macrophage phenotype regulation.

**Fig. 5:**
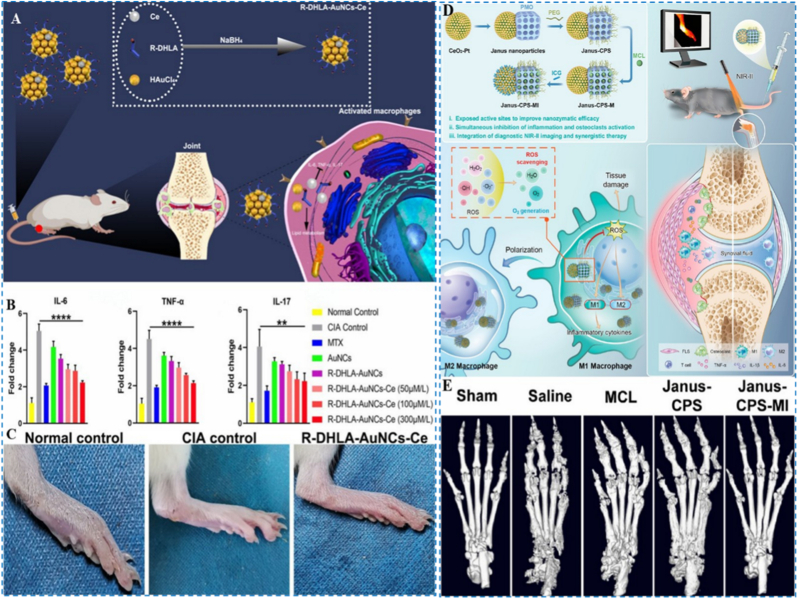
(A) Preparation workflow of R-DHLA-AuNCs-Ce with a schematic overview of RA-related mechanisms of action. (B) Quantification of IL-6, TNF-α, and IL-17 among different treatment groups. (C) Images of the right lower limb. Reproduced with permission from Ref. [75]. Copyright American Chemical Society 2022. (D) Preparation scheme of the Janus nanoplatform (Janus-CPS-MI) and its dual functionality in early RA diagnosis and combination therapy. (E) Three-dimensional micro-CT reconstructions of hind paws in the noted groups. Reproduced with permission from Ref. [77]. Copyright American Chemical Society 2023.

As nanotechnology continues to advance, delivery methods and multifunctional synergy have emerged as key strategies for enhancing therapeutic efficacy. Lin et al. developed a gold nanocluster (R-DHLA-AuNCs-Ce) containing (R)-dihydrothioctic acid (R-DHLA) and Ce for the relief of early RA (Fig. 5A) [75]. Treatment with R-DHLA-AuNCs-Ce significantly reduced IL-6, TNF-α, and IL-17 levels in in-vitro assays (Fig. 5B). After co-culturing the nanoparticles with macrophages, ROS staining results revealed a significant reduction in intracellular ROS levels, suggesting their excellent antioxidant capacity. Additionally, this nanomaterial system enhanced macrophage lipid metabolism, with Oil Red O staining indicating increased lipid accumulation. These findings suggest that normalization of lipid metabolism may suppress the release of pro-inflammatory mediators. Consistent with this, in vivo studies showed that R-DHLA-AuNCs-Ce markedly attenuated disease manifestations in collagen-induced arthritis (CIA) rats and outperformed the standard agent MTX. Together, the data highlight a promising avenue for early RA intervention (Fig. 5C).

Building on the Ce-based nanomaterial system, researchers have further explored multifunctional and diagnostic-therapeutic integrated dynamic platforms. Xu et al. proposed MnO_2_-motor particles driven by environmental H_2_O_2_, combining CeO_2_'s ROS scavenging function with MnO_2_'s efficient oxygen-producing capability [76]. The platform mitigates inflammatory and hypoxic conditions, drives macrophage repolarization from M1 to M2, and exhibits pronounced anti-inflammatory and osteoprotective efficacy in animal models. Liu et al. designed a Janus nanoplatform (Janus-CPS-MI) that enables concurrent early diagnosis and synergistic therapy for RA (Fig. 5D) [77]. One side of the platform consists of CeO_2_-Pt nanocatalysts, while the other side is composed of periodic mesoporous organic silica. The Janus configuration increases the exposure of catalytic sites, markedly boosting ROS-scavenging performance. The silica subunit is loaded with the natural anti-osteoclastogenic active molecule micheliolide, which works synergistically with the anti-inflammatory action of the nanoenzyme [78]. Both in vitro and in vivo evaluations verified that the platform confers strong anti-inflammatory activity and protects against bone destruction in RA (Fig. 5E). Additionally, Janus-CPS-MI can load ICG for NIR-II fluorescence imaging, thereby enhancing the sensitivity and accuracy of RA lesion detection.

Additionally, Xia et al. integrated Ce/manganese oxide nanoenzymes with MTX into a microneedle delivery platform to achieve transdermal targeted drug delivery, synergistically clearing ROS and regulating macrophage polarization. Animal experiments demonstrated its high efficacy in alleviating inflammation, along with excellent biological safety [79]. Zhang et al. designed microwave-sensitive metal-organic framework materials that combine the multi-enzyme activity of CeO_2_ with microwave-driven multi-synergistic effects [80]. The platform augments both ROS elimination and oxygen generation, while exhibiting potent anti-inflammatory activity and favorable safety in animal studies. This series of delivery and functional innovations reflects the trend of Ce-based systems evolving toward “multi-modal, precise, and controllable” strategies.

#### 2.1.2 Non-cerium metal nanozyme-based ROS-scavenging nanosystems

**Fig. 6:**
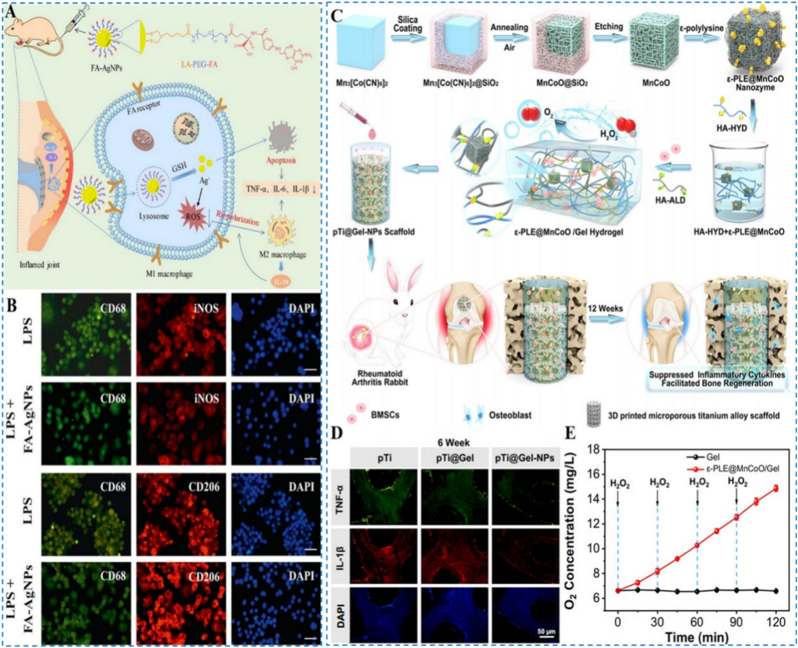
(A) Preparation workflow of FA-Ag nanoparticles with a schematic overview of their RA-related mechanisms of action. (B) Immunofluorescence staining of CD68, iNOS and CD206, and nuclei on macrophages without or with treatment of FA-Ag nanoparticles. Reproduced with permission from Ref. [81]. Copyright Elsevier 2021. (C) Diagram of the preparation process for a nanozyme-reinforced self-protecting hydrogel and its use in improving prosthetic interface osseointegration for RA treatment. (D) Immunofluorescence staining of TNF-α and IL-1β in bone surrounding the scaffolds. (E) Demonstration of sustained catalytic O_2_ production from ε-PLE@MnCoO/Gel upon successive H_2_O_2_ dosing. Reproduced with permission from Ref. [83]. Copyright American Chemical Society 2023.

Beyond Ce, other metal ions, including silver, iron, cobalt, and platinum, exhibit distinctive redox chemistry and enzyme-mimetic activities that can be harnessed to modulate oxidative stress in RA. Nanoscale platforms built on these ions not only efficiently eliminate ROS within joint tissues but also remodel the inflammatory microenvironment through complementary mechanisms, thereby promoting joint repair and tissue regeneration. Recent studies have emphasized multifunctional antioxidant synergy, inflammation-targeted delivery, and favorable biocompatibility. Yang et al. developed silver nanoparticles functionalized with FA (FA-Ag NPs), thereby combining innate ROS-scavenging properties with FA receptor-mediated macrophage homing (Fig. 6A) [81]. The platform markedly lowered intracellular ROS in macrophages and drove repolarization from the pro-inflammatory M1 state to an anti-inflammatory M2 phenotype (Fig. 6B), with broad suppression of inflammatory mediators; concordant in-vitro and in-vivo data demonstrated strong anti-inflammatory efficacy alongside favorable biocompatibility and safety. By integrating active and passive targeting, this strategy offers a novel, safe, and efficient design paradigm for metal-based nanotherapeutics in RA.

In addition, Yang and colleagues produced iron hydroxide nanomaterials with nanozyme properties, catalytically converting H_2_O_2_ into O_2_ to quench ROS and promote a shift in macrophages from pro-inflammatory M1 toward M2 phenotypes [82]. Demonstrating significant catalase-like reactivity across physiologically relevant pH, the nanoparticles suppress inflammation and preserve bone-marrow-derived mesenchymal stem cells, thereby aiding joint restoration; animal work confirmed reductions in swelling and bone damage. Furthermore, Zhao et al. combined manganese-cobalt oxide nanozymes with a dynamically cross-linked hyaluronic-acid hydrogel to construct a composite system (ε-PLE@MnCoO/Gel) endowed with excellent self-healing capacity and biocompatibility (Fig. 6C) [83]. This platform reduced inflammatory cytokines in RA model animals (Fig. 6D), enhanced O_2_ generation and osteointegration (Fig. 6E), and contributed to bone-tissue regeneration and cartilage protection, demonstrating strong application potential.

Notably, Guo et al. developed a bovine serum albumin-bilirubin-platinum nanozyme platform that couples the radical-scavenging activity of bilirubin with the CAT-like function of platinum nanoparticles [84]. The platform not only removes ROS efficiently while sustaining O_2_ generation, but also reprograms cellular metabolism to drive macrophage repolarization from M1 to the anti-inflammatory M2 phenotype, thereby synergistically attenuating RA inflammation. In vivo, treated animals exhibited reduced M1 macrophage abundance within the synovium, enhanced M2 polarization, and no evidence of hepatorenal toxicity, indicating an excellent safety profile. Taken together, non-cerium metal ion–based nanostructures, by integrating multi-enzyme–mimetic activities, targeted delivery, and modulation of the inflammatory microenvironment, provide expanded antioxidant strategies and therapeutic opportunities for RA, while laying a conceptual and practical foundation for next-generation intelligent metal-based nanotherapeutics with multi-mechanistic synergistic effects.

#### 2.1.3 Polydopamine (PDA)-Based ROS-scavenging nanosystems

**Fig. 7:**
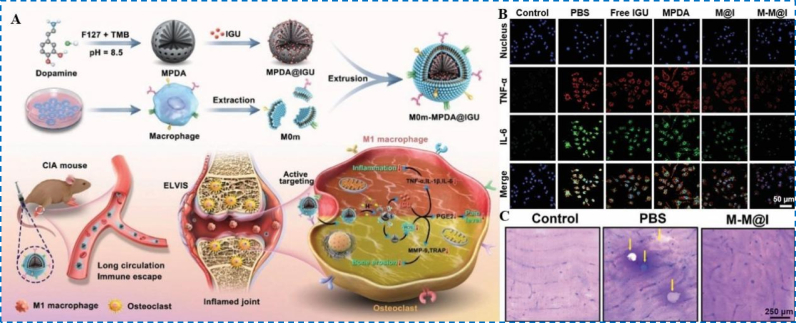
(A) Schematic depiction of a pathology-specific biomimetic multifunctional nanoplatform for RA-targeted therapy. (B) Immunofluorescence images of TNF-α and IL-6 expression in RAW 264.7 macrophages under LPS stimulation. (C) Bovine bone slices stained with toluidine blue. Reproduced with permission from Ref. [87]. Copyright Wiley 2023. (For interpretation of the references to colour in this figure legend, the reader is referred to the Web version of this article.)

Polydopamine (PDA) exhibits excellent ROS scavenging capacity owing to its abundant phenolic hydroxyl and amino functional groups [85,86]. In recent years, researchers have harnessed PDA's antioxidant properties, surface modification versatility, and self-assembly capability to construct novel nanoplatforms, achieving innovations that extend from passive scavenging to active targeted delivery. These strategies provide promising avenues for the precise treatment of RA. Xu et al. designed a biomimetic multifunctional nanomedicine (M-M@I) using mesoporous PDA as a carrier to load Iguratimod, while coating the exterior with macrophage membranes to confer active recognition and localization to inflammatory sites (Fig. 7A) [87]. This system enabled precise targeting of RA lesions in vivo and efficiently eliminated ROS within macrophages, thereby synergistically downregulating multiple pro-inflammatory factors (Fig. 7B). Animal experiments demonstrated that the M-M@I nanodrug significantly alleviated cartilage destruction and bone erosion in mouse joints (Fig. 7C), exhibiting potent bone-protective and anti-inflammatory effects. These findings validate the therapeutic potential of PDA-based nanodrug systems in RA treatment.

**Fig. 8:**
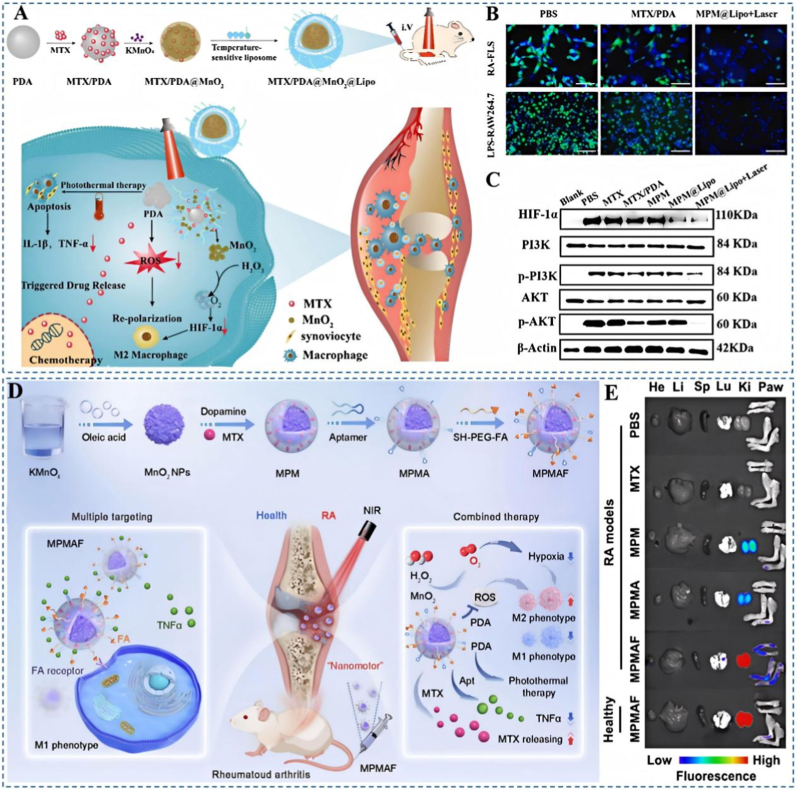
(A) Overview of MPM@Lipo construction and the proposed mechanisms underlying anti-RA activity. (B) Fluorescent microscopy of intracellular ROS levels. (C) Protein expression of HIF-1α, PI3K, p-PI3K, AKT and p-AKT. Reproduced with permission from Ref. [88]. Copyright Elsevier 2024. (D) The designing of multifunctional MPMAF nanoplatforms for dual targeting and combined therapy of RA. (E) Ex vivo fluorescent images of the principal organs and the four paws in RA mice after MPMAF administration. Reproduced with permission from Ref. [89]. Copyright American Chemical Society 2025.

To further extend the multifunctionality of the PDA platform, Fu et al. engineered multifunctional liposome-encapsulated PDA nanoparticles (MPM@Lipo) that integrate chemotherapy, photothermal therapy (PTT), and oxygenation [88]. Upon laser irradiation, the system locally elevates temperature to ablate inflammatory cells while PDA efficiently scavenges ROS, thereby markedly improving hypoxia and the inflammatory microenvironment within the joint cavity (Fig. 8A). In the MPM@Lipo + laser group, ROS levels were significantly reduced (Fig. 8B), HIF-1α and the PI3K/AKT pathway were concomitantly inhibited (Fig. 8C), and macrophages shifted toward M2 polarization; notable biosafety further supports the expanded therapeutic scope of PDA-enabled multimodal systems. Li et al. further designed a multifunctional drug-delivery nanoplatform employing a dual-targeting strategy in which FA and a TNF-α-specific aptamer synergistically guide nanoparticle accumulation at RA lesions. The platform surface is coated with PDA to co-encapsulate MTX and manganese dioxide, enabling potent ROS scavenging, relief of hypoxia, and repolarization of pro-inflammatory M1 macrophages toward M2, thereby improving the inflammatory microenvironment (Fig. 8D) [89]. In vivo fluorescence imaging confirmed precise targeting of RA joint lesions (Fig. 8E), and combined in vitro/in vivo assays verified the platform's ROS-scavenging, hypoxia-alleviating, and macrophage-reprogramming functions, achieving effective RA control. This study integrates targeted delivery, microenvironment modulation, and immune remodeling to build a multifunctional nanoplatform that couples anti-inflammatory therapy with precise drug delivery, offering a new strategy for RA combination therapy. Additionally, Luo et al. constructed a multi-component therapeutic by integrating the nitric oxide (NO) donor S-nitrosoglutathione, the ROS scavenger PDA, the targeting ligand sulfated dextran, and the NO-generating catalyst Cu^2+^ [90]. This system simultaneously suppresses ROS accumulation, augments NO levels, and blocks NF-κB signaling, thereby limiting pro-inflammatory macrophage polarization; both cellular and animal studies showed robust mitigation of joint inflammation, reductions in joint damage and bone erosion, and significant improvements in clinical scores and paw thickness, demonstrating strong anti-RA efficacy.

As demonstrated by prior studies, PDA-based nanomaterials not only exhibit intrinsic antioxidant activity that efficiently scavenges ROS, but also serve as versatile platforms that can be integrated with advanced drug-delivery, targeting, and multimodal therapeutic strategies to concurrently enhance selectivity, therapeutic synergy, and safety in RA management. Continued innovation in these materials is poised to expand the technological repertoire and mechanistic framework underpinning precise and personalized RA therapy.

#### 2.1.4 Polyphenolic compound-based ROS-scavenging nanosystems

Polyphenolic compounds are a broad class of plant-derived molecules (e.g., tea polyphenols and quercetin) whose dense phenolic hydroxyl groups confer potent anti-oxidant and anti-inflammatory effects [91]. In the context of RA, these compounds directly neutralize ROS via hydrogen-atom transfer and single-electron transfer, thereby terminating oxidative chain reactions and effectively limiting oxidative damage to joint tissues [92]. In addition, by sequestering transition metal ions such as Fe^2+^ and Cu^+^, polyphenols inhibit Fenton chemistry and thereby reduce the generation of highly reactive hydroxyl radicals (·OH) [93]. Recently, nanoplatforms constructed from or functionalized with polyphenolic compounds have demonstrated substantial advantages in ROS scavenging, suppression of inflammatory signaling, and preservation of joint structure, offering new avenues for precision antioxidant therapy in RA [94].

Lu et al. synthesized rosmarinic acid nanoparticles (RNPs) via oxidation-oligomerization-driven self-assembly using rosmarinic acid as the precursor [95]. In cell-based assays, RNPs reduced intracellular ROS and upregulated the enzymatic activities of SOD and CAT, demonstrating robust antioxidant cytoprotection. Transcriptomic profiling further showed that RNPs modulate core pathways related to immunity, inflammation, and cytokine signaling, with effective suppression of TNF and JAK-STAT networks. In animal models, RNPs curtailed synovial hyperplasia and angiogenesis, promoted M1 to M2 macrophage repolarization, reduced IL-1β and IL-6, and increased IL-10, indicating effective regulation of reactive oxygen and nitrogen species and mitigation of inflammation.

To optimize delivery, Lin et al. engineered chlorogenic acid (CA) loaded liposomes and further enhanced their stability and targeting by embedding them within a sodium alginate nanogel [96]. CA is a natural anti-inflammatory polyphenol, and liposomal encapsulation amplified its ROS-scavenging and anti-inflammatory activities. This nanogel-liposome composite not only efficiently scavenged ROS and downregulated intracellular pro-inflammatory mediators (e.g., TNF-α), but also improved systemic circulation and cellular uptake, thereby enabling precise, lesion-targeted distribution. In an AIA rat model, CA liposomes significantly attenuated joint swelling and inflammatory responses while maintaining an excellent safety profile, highlighting how delivery-platform optimization can potentiate the anti-inflammatory efficacy of polyphenols.

**Fig. 9:**
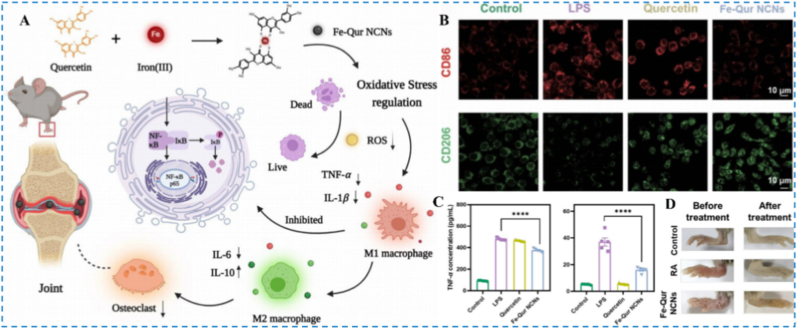
(A) Ultrasmall iron-quercetin nanocomplex demonstrating antioxidant effects and macrophage regulation relevant to RA. (B) Confocal visualization of M1 and M2 macrophage states. (C) Quantification of TNF-α and IL-1β released by RAW 264.7 cells following different interventions. (D) Images of hind-paw morphology. Reproduced with permission from Ref. [97] Copyright Elsevier 2023.

The coordination of polyphenols with metal ions has emerged as a promising route to novel antioxidant platforms. Han et al. combined Fe (III) with quercetin to generate ultrasmall iron-quercetin natural coordination nanoparticles (Fig. 9A), which efficiently scavenged ROS and significantly inhibited NF-κB signaling, thereby driving M1 to M2 macrophage repolarization (Fig. 9B) [97]. In addition, it downregulated pro-inflammatory phenotype markers (Fig. 9C), and reduced joint swelling and composite inflammation scores (Fig. 9D), demonstrating superior therapeutic efficacy. In parallel, Yi et al. fabricated polyphenol-based nanoparticles using epigallocatechin gallate (EGCG) and 4-aminophenylboronic acid pinacol ester, further loading dexamethasone (DEX) to construct a multimodal anti-inflammatory delivery system [98]. This platform exploits the ELVIS effect, extravasation through leaky vasculature followed by inflammatory cell-mediated sequestration, to passively target inflamed joints [99]. EGCG and the boronate ester synergistically scavenge ROS and suppress macrophage activation, while DEX complements these actions by downregulating inflammation-related genes; concordant in vitro and in vivo data show marked reductions in ROS and inflammatory mediators, outperforming EGCG monotherapy, with no appreciable systemic toxicity.

In summary, nanoplatforms derived from polyphenolic hydroxy compounds exhibit favorable biosafety profiles and multitarget synergistic activity in RA therapy. Through rational structural innovation and delivery-platform optimization, these materials enable efficient ROS scavenging, modulation of the inflammatory microenvironment, and protection of joint tissues.

#### 2.1.5 Prussian blue (PB) nanozyme-based ROS-scavenging nanosystems

**Fig. 10:**
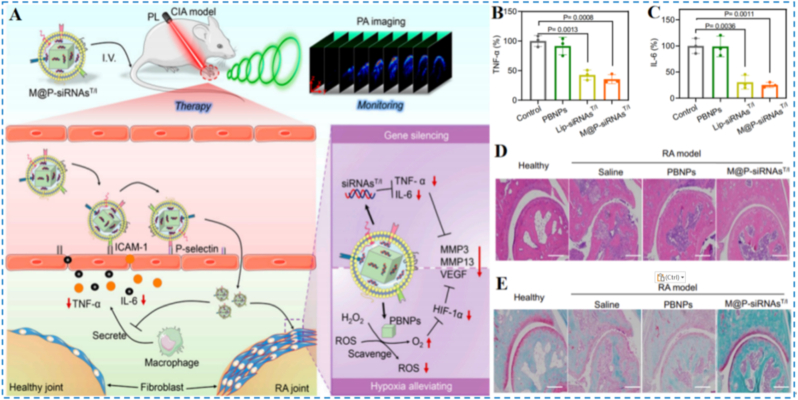
(A) Overview schematic illustrating M@P-siRNAs^T/I^ as a PA imaging-guided platform for RA treatment. (B and C) The level of TNF-α and IL-6. (D) Representative H&E images. (E) Representative safranin-o staining images. Reproduced with permission from Ref. [107]. Copyright National Academy of Sciences of the United States of America 2022.

Among antioxidant nanosystems, Prussian blue nanoparticles (PBNPs) have garnered considerable attention for RA therapy owing to their enzyme-mimetic activities and excellent biocompatibility [100,101]. Acting as enzyme mimics of catalase and peroxidase (POD), PBNPs catalytically decompose excess ROS within the arthritic microenvironment and generate O_2_ from H_2_O_2_, thereby alleviating hypoxia, dampening inflammatory responses, and protecting cartilage [102]. Recently, researchers have integrated PB with advanced modalities, including gene regulation, targeted delivery, and multimodal imaging, substantially broadening its application landscape in precision RA therapy [[103], [104], [105], [106]]. For instance, Chen et al. engineered a biomimetic vesicle platform derived from macrophage membranes (M@PsiRNAsT/I) that integrates small interfering RNAs, PBNPs, and near-infrared photoacoustic imaging (Fig. 10A) [107]. Leveraging the homotypic targeting of macrophage membranes, the platform efficiently delivers siRNAs and PBNPs to arthritic lesions, silences TNF-α and IL-6, clears ROS, and simultaneously enables real-time monitoring of therapeutic efficacy via near-infrared photoacoustic imaging. In vitro, M@PsiRNAsT/I markedly reduced pro-inflammatory mediator expression in macrophages (Fig. 10B and C); in vivo, treated mice exhibited diminished synovial hyperplasia and inflammatory cell infiltration (Fig. 10D) and improved cartilage integrity (Fig. 10E), without systemic toxicity. By uniting gene regulation, ROS elimination, and imaging guidance, this approach provides a highly integrated route for precision RA treatment.

To further enhance multi-mechanism synergy, Lin et al. constructed HA@M@PB@SIN nanoparticles that unite hyaluronic acid (HA) and macrophage-membrane dual modification for targeted synovial delivery with PB nanozyme catalysis and drug cargo [108]. PBNPs mimic POD activity to markedly eliminate ROS within inflamed joints and catalyze O_2_ generation to relieve synovial hypoxia, while the loaded sinomenine hydrochloride further suppresses inflammation, including through NF-κB pathway inhibition. This dual-ligand surface engineering substantially improves accumulation in inflamed synovium and joints, and both in vitro and in vivo studies show broad down-regulation of pro-inflammatory mediators, reduced synovial hyperplasia and inflammatory infiltration, and significant cartilage preservation, with no detectable toxicity to major organs, indicating favorable biocompatibility and safety. Collectively, PB-based nanosystems have emerged as a key avenue for precision RA nanotherapy owing to their potent ROS-scavenging capacity, complementary mechanistic actions, and excellent targeting performance; looking ahead, PB-centered composite platforms are well positioned to expand into intelligent inflammation regulation, multi-therapy combinations, and image-guided interventions.

#### 2.1.6 DNA origami-based ROS-scavenging nanosystems

**Fig. 11:**
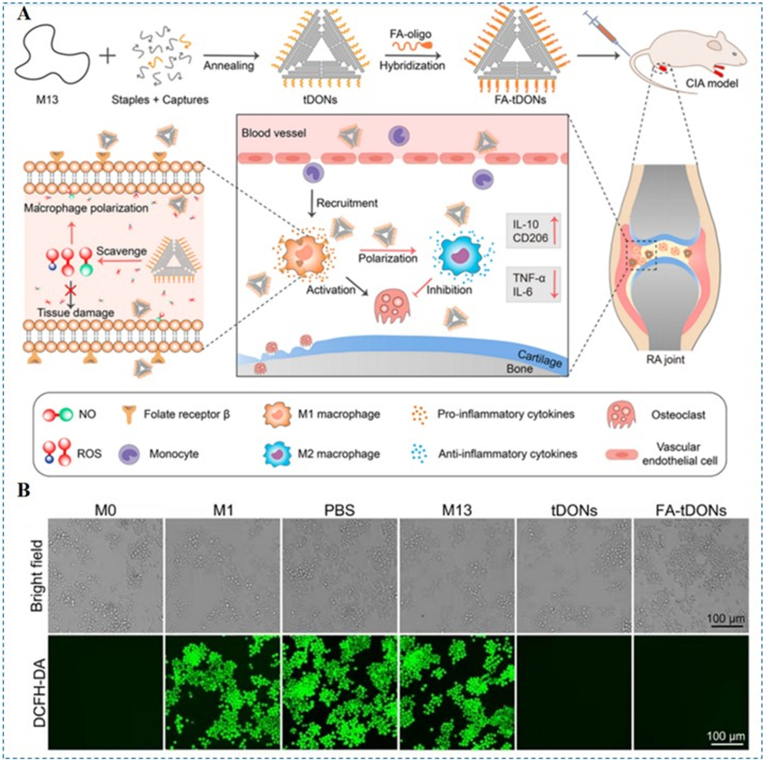
(A) The design strategy and mechanism of FA-tDONs nanomedicine for RA treatment. FA-tDONs are constructed from triangular DNA origami coupled with folate (FA), a targeting moiety for M1 macrophages. By co-eliminating ROS and NO, they actively engage M1 cells and drive polarization toward M2, attenuating inflammation, limiting osteoclastogenesis, and alleviating RA advancement. (B) Fluorescence imaging of ROS scavenging efficacy within M1 macrophages for different DNA materials using DCFH-DA (green) as a probe. Reproduced with permission from Ref. [109]. Copyright American Chemical Society 2023. (For interpretation of the references to colour in this figure legend, the reader is referred to the Web version of this article.)

Ma et al. designed a triangular DNA origami nanostructure with targeting capability for RA therapy (Fig. 11A) [109]. The platform uses FA ligands to selectively target M1 macrophages, thereby enhancing cellular uptake within inflamed joints. Upon internalization, the DNA nanostructures efficiently scavenge ROS (Fig. 11B). In cellular assays, the treatment decreased the M1 macrophage marker iNOS by 74 % and raised the M2 marker CD206 by 2.8-fold, thereby promoting M1 to M2 repolarization. In vivo, AIA mice treated with the nanomedicine showed effective relief of arthritis symptoms without obvious histopathological abnormalities in major organs, indicating favorable biosafety. Mechanistically, DNA origami nanostructures can participate directly in redox reactions with ROS [110]. The π-stacked nucleobases, especially guanine, are prone to oxidation and can act as sacrificial electron donors that quench radical species, thereby buffering local ROS. In addition, the densely packed phosphate backbone and nucleobase arrays provide multiple binding sites for redox-active metal ions, which can reduce Fenton-type reactions and secondary hydroxyl radical generation. Together, these features endow DNA origami-based platforms with both physical shielding and chemical scavenging capacity toward ROS, contributing to macrophage repolarization and attenuation of inflammatory cascades in RA.

#### 2.1.7 α-Tocopherol succinate (α-TOS)–based ROS-scavenging nanosystems

**Fig. 12:**
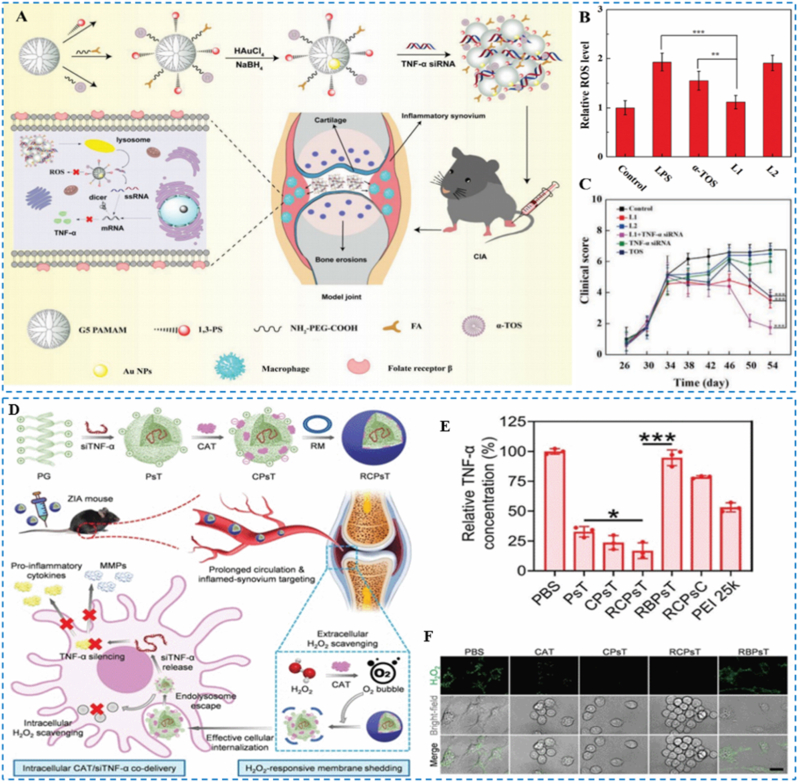
(A) Schematic of α-TOS-modified dendrimer-encapsulated nanoparticles complexed with TNF-α siRNA polyplexes for RA therapy. (B) Flow cytometry assay of the ROS scavenging capacity of different materials. (C) Changes in clinical scores over time before and after treatments. Reproduced with permission from Ref. [111]. Copyright Wiley 2020. (D) Diagram depicting TNF-α knockdown coupled with H_2_O_2_ elimination for RA intervention. (E) TNF-α protein levels in RAW 264.7 cells. (F) Images displaying ROS level. Reproduced with permission from Ref. [112]. Copyright Wiley 2023.

Li et al. developed a multifunctional nanoplatform that co-delivers the antioxidant α-TOS and anti-TNF-α siRNA to macrophages by employing generation-5 poly(amidoamine) based gold dendrimer-encapsulated nanoparticles for RA therapy (Fig. 12A) [111]. Flow cytometry and qRT-PCR analyses showed efficient scavenging of intracellular ROS in macrophages (Fig. 12B) and downregulated expression of oxidative-stress–related genes, including HO-1, SOD2, and NOX2. In the CIA mouse model, treatment markedly reduced clinical arthritis scores and paw swelling (Fig. 12C), while micro-CT and histology demonstrated improved bone microarchitecture and synovial integrity, with diminished bone-volume loss, reduced inflammatory cell infiltration and synovial hyperplasia, and preserved cartilage. H&E staining indicated no overt toxicity in major organs. Together, these data suggest that the platform mitigates RA pathology through synergistic antioxidant and anti-inflammatory mechanisms and exhibits favorable biosafety, underscoring its translational potential.

#### 2.1.8 Catalase (CAT)-Based ROS-scavenging nanosystems

Shan et al. developed a biomimetic nanocomplex composed of a macrophage-membrane cloak encapsulating a cationic helical peptide, anti-TNF-α siRNA (siTNF-α), and CAT for RA therapy (Fig. 12D) [112,113]. In a ROS-rich milieu, CAT catalyzes the decomposition of H_2_O_2_ to generate O_2_, which triggers membrane detachment and exposes the cationic inner core, thereby enhancing membrane penetration and uptake by synovial macrophages. Core delivery of siTNF-α achieves TNF-α gene silencing, while CAT concurrently scavenges ROS. Together, these actions synergistically suppress inflammation and oxidative stress and promote repair of the bone microenvironment. The macrophage membrane confers prolonged circulation and inflammation-homing capability. In vitro, H_2_O_2_ stimulation promoted efficient cellular uptake, significant downregulation of TNF-α expression, and ROS scavenging, thereby alleviating joint inflammation (Fig. 12E–F). In vivo histopathology and scoring further indicated robust therapeutic efficacy with favorable biosafety.

In summary, ROS-scavenging nanosystems integrate metal-based (e.g., cerium, silver ions) and non-metal-based scavenging components to reduce pathological ROS levels and inhibit abnormal proliferation of inflammatory cells. The components, cell models, and animal models of the ROS-scavenging nanosystems are summarized in Table 1. Metal ions achieve catalytic ROS scavenging through reversible valence state switching, while non-metal components neutralize ROS via potent antioxidant capacity, thereby significantly alleviating joint swelling and inflammatory responses. This system holds both significant mechanistic research value and clinical application potential in RA treatment.

**Table 1:** ROS-scavenging nanosystems: agents and RA models.

Agents	Cell Models	Animal Models	Ref.
Metal ions	RAW 264.7 cells; Bone marrow-derived macrophages; Bone marrow-derived mesenchymal stem cells; L929 Cells	AIA SD rats; AIA BALB/c mice; CIA DBA/1 mice; New Zealand white rabbits	[64,71,75,77,[79], [80], [81], [82], [83], [84]]
PDA	RAW 264.7 cells	AIA SD rats; CIA DBA/1 mice	[[87], [88], [89], [90]]
Polyphenol	RAW 264.7 cells; Human promyelocytic leukemia cells	AIA SD rats; CIA SD rats	[[95], [96], [97]]
PB	RAW 264.7 cells; L929 cells; HFLS cells	AIA SD rats; CIA DBA/1 mice	[107,108]
DNA	RAW 264.7 cells	CIA DBA/1 mice	[109]
Tocopheryl succinate	RAW 264.7 cells	CIA DBA/1 mice	[111]
CAT	RAW 264.7 cells	None	[112]

### 2.2 ROS-responsive nanosystems for RA treatment

**Fig. 13:**
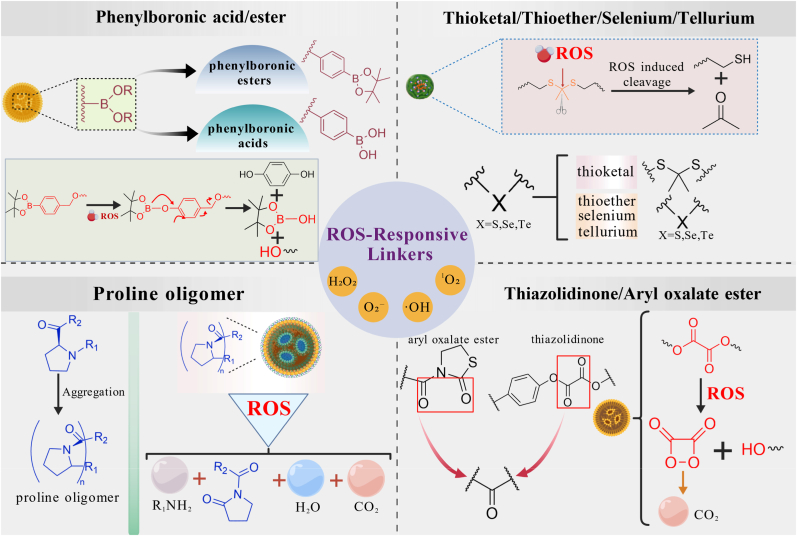
Representative ROS-responsive sites and their underlying response mechanisms.

In RA therapeutics, the ROS-responsive mechanism has become a central paradigm for intelligent nanomedicine design, as many materials are highly sensitive to ROS and can enable “switch-like” drug delivery via chemical-bond scission, conformational rearrangement, or surface-charge conversion [[114], [115], [116], [117], [118]]. Such smart nanosystems markedly improve targeting and spatiotemporal control of delivery and permit dose tuning through dynamic regulation of release kinetics, thereby minimizing off-target toxicity and systemic adverse effects. Design strategies include embedding ROS-labile motifs (e.g., thioethers, thioketals, boronic esters, sulfoxides/sulfones) into polymer backbones or side chains, or leveraging redox-reactive matrices to trigger carrier disassembly or pore opening upon ROS exposure (Fig. 13). The ROS-responsive nanosystems reviewed in this section rely solely on ROS as the trigger for drug release and do not themselves modulate ROS levels; platforms that both respond to and deplete ROS are discussed in the subsequent section.

**Fig. 14:**
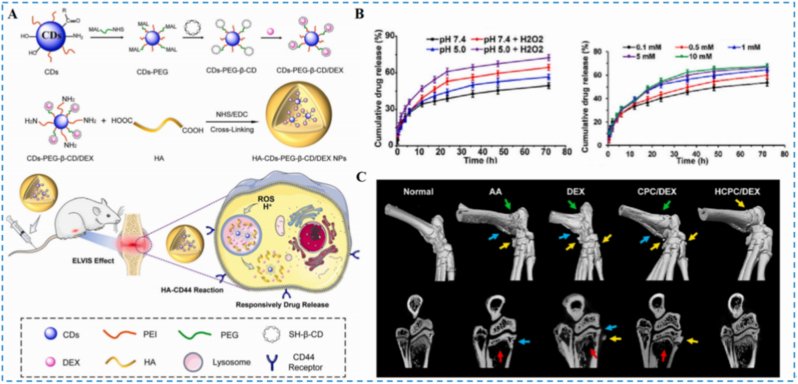
(A) Schematic depiction of the preparation of nanomaterials and their applications in RA treatment. (B) The release of environmentally responsive drugs. (C) The representative visual 3D images of ankle joints. Reproduced with permission from Ref. [119]. Copyright Elsevier 2023.

Wang et al. developed a DEX-loaded targeted delivery platform based on HA and β-cyclodextrin for RA (Fig. 14A) [119]. In ROS-rich and mildly acidic RA microenvironments, oxidative degradation of HA induces carrier disassembly and accelerates DEX release. In vitro, the release rate increased with rising H_2_O_2_ concentrations, confirming ROS responsiveness (Fig. 14B). Surface HA engages CD44 receptors on M1 macrophages to enable active targeting, and the platform effectively downregulated pro-inflammatory cytokines, inhibited M1 polarization, and exhibited low cytotoxicity. In vivo, treated animals showed significant reductions in joint swelling and inflammation; micro-CT corroborated therapeutic benefit, and overall biosafety was favorable (Fig. 14C). This work leverages HA redox responsiveness to enhance the targeting and safety profile of DEX for RA therapy.

Chen and colleagues developed a nanocarrier responsive to both pH and ROS, enabling CD44-mediated co-delivery with staged, on-demand release of tofacitinib followed by glucosamine to achieve synergistic treatment of RA [120]. Exploiting the high CD44 expression on M1 macrophages, the platform achieves active targeting via CD44-chondroitin sulfate affinity [121]. A pH-labile imine (Schiff-base) linkage cleaves under acidic conditions to preferentially release glucosamine for cartilage repair, whereas in ROS-rich milieus oxidation-triggered conversion of methylsulfonylpropylamine drives nanoparticle disassembly and the concurrent release of tofacitinib/glucosamine. Together, the two agents drive macrophage repolarization from M1 to M2 and dampen inflammation. In vitro, the nanoparticles reduced p-p65, decreased the proportion of the M1 marker CCR7, and increased the M2 marker CD206, evidencing strong anti-inflammatory activity. In the CIA model, they yielded the lowest arthritis scores with favorable in vivo safety.

In summary, ROS-responsive nanomedicine delivery systems utilize ROS-sensitive carrier materials to load therapeutic agents, enabling their degradation or conformational changes at sites of inflammation where ROS levels are elevated. This facilitates targeted drug release, effectively reducing off-target toxicity and systemic adverse reactions while enhancing therapeutic safety. Such systems primarily employ ROS as a release trigger signal without directly regulating ROS levels; their therapeutic efficacy relies chiefly on the pharmacological effects of the loaded drugs. This delivery strategy demonstrates significant application value and research potential for targeted therapy in RA.

### 2.3 ROS-responsive and scavenging composite nanosystems for RA treatment

The pathological microenvironment of RA lesions is characterized by elevated ROS, mild acidosis (reduced pH), and localized temperature increases [122]. Building on these features, researchers have designed “switch-and-scavenge” nanosystems that embed dynamic chemistries highly sensitive to ROS, pH, or temperature to trigger targeted release of therapeutic or antioxidant cargos [32]. These platforms not only release anti-inflammatory or antioxidant agents under high-ROS, low-pH, or externally stimulated conditions (e.g., microwave irradiation), but also selectively scavenge ROS at inflamed sites, interrupt pro-inflammatory signaling, restore redox homeostasis, enhance drug accumulation within affected joints, and minimize systemic off-target exposure [[123], [124], [125], [126], [127], [128]]. By coupling microenvironment recognition with precise, controllable release kinetics, ROS-responsive nanosystems offer a promising route to targeted, spatiotemporally controlled RA therapy. Recent studies in RA models have validated their marked efficacy, opening avenues for individualized and precision treatment [[129], [130], [131]].

#### 2.3.1 ROS-responsive and scavenging nanosystems (single stimulus)

In response to the pathological characteristic of elevated ROS expression within the RA joint microenvironment, mono-stimuli-responsive nano-delivery systems have emerged. These systems typically introduce dynamic chemical bonds, such as thiols and thioethers, as ROS- sensitive switches. By combining functional molecules, including anti-inflammatory drugs (e.g., DEX) and antioxidant enzymes (e.g., CAT), they achieve on-demand drug release specifically within high-ROS environments, thereby enhancing therapeutic precision [132,133]. By exclusively responding to ROS signals, these nanosystems effectively circumvent the off-target risks associated with multi-target interventions. This characteristic renders them suitable for the precise modulation of single pathological pathways, providing an important tool and indicating a development direction for intelligent, individualized RA therapy.

**Fig. 15:**
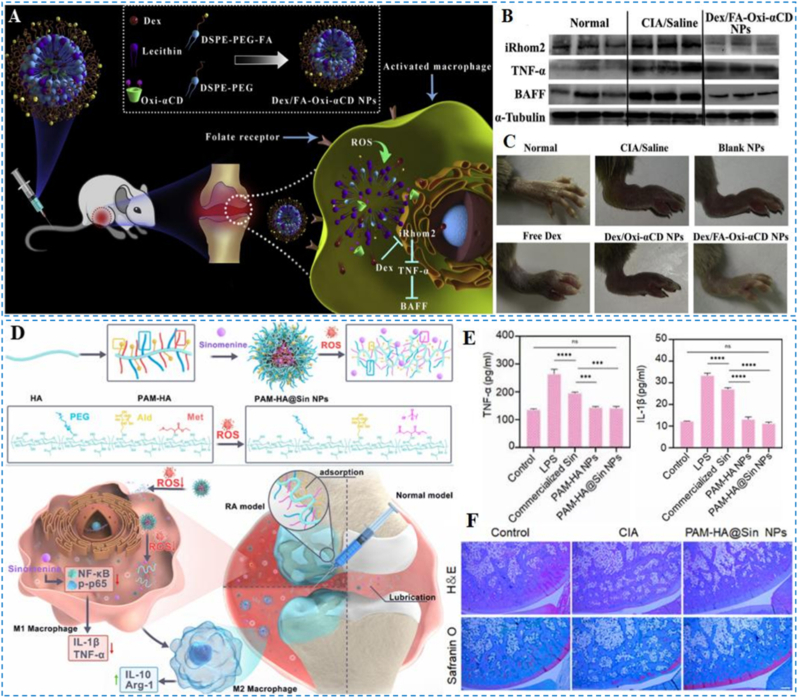
(A) Schematic depiction of Dex-loaded, ROS-triggered nanoparticles designed for RA-targeted therapy. (B) Expression profiles of iRhom2, TNF-α, and BAFF in joints. (C) Representative hind-leg photographs from the indicated treatment groups. Reproduced with permission from Ref. [132]. Copyright Elsevier 2020. (D) Schematic of PAM-HA@Sin nanoparticle fabrication and the corresponding ROS-responsive process. (E) Quantification of IL-1β and TNF-α. (F) Histopathology of arthritic tissues assessed by H&E and Safranin O staining. Reproduced with permission from Ref. [144]. Copyright American Chemical Society 2023.

Ni et al. developed a FA-targeted, ROS-responsive nano-delivery system for precision RA therapy (Fig. 15A) [132]. The platform employs ROS-responsive 4-phenylboronic acid pinacol ester-conjugated cyclodextrin biomaterials (Oxi-αCD) derivatives as carriers to load DEX, while FA modification enhances targeting to inflamed sites. Within ROS-rich inflammatory microenvironments, the Oxi-αCD derivatives undergo oxidative disassembly, enabling synchronous ROS scavenging and controlled DEX release [134]. The released DEX synergistically attenuates inflammation [135]. In vitro, the system markedly reduced intracellular ROS. In the CIA mouse model, the nanoplatform alleviated paw swelling, lowered arthritis scores, and downregulated inactive rhomboid-like protein 2 (iRhom2), TNF-α, and B-cell Activating Factor (BAFF) expression in joint tissues (Fig. 15B and C), with histology confirming reduced synovial hyperplasia and inflammatory cell infiltration.

Song et al. designed ROS-responsive liposomes composed of dimeric thiophospholipids and employed FA modification to precisely target M1 macrophages [136]. Under high ROS conditions, the dimeric thioether lipids (di-S-PC) are cleaved, which both scavenges ROS and triggers the controlled release of DEX, thereby downregulating iRhom2 and other inflammatory mediators [137]. Both in vitro and in vivo studies demonstrated significant attenuation of RA inflammation with a favorable safety profile. Advancing platform innovation, Li et al. developed a ROS-responsive system based on artesunate prodrug micelles that, via CD44-mediated targeting, undergo oxidative cleavage in high-ROS milieus to co-release artesunate and DEX [[138], [139], [140]]. The co-delivery regimen enhanced ROS neutralization and anti-inflammatory efficacy, promoted macrophage switching from M1 to M2, and robustly inhibited HIF-1α/NF-κB signaling in vivo, thereby lowering inflammation and protecting key organs.

Zhou et al. extended the ROS-responsive strategy by employing HA-modified nanomicelles to co-deliver CeO_2_ nanozymes and rhein, incorporating a thioketal linker to confer ROS-triggered disassembly and thereby achieving efficient ROS scavenging [[141], [142], [143]]. This platform increased the proportion of M2 macrophages and reduced ROS levels in both cellular and animal models, with gradual metabolic clearance of the inorganic component and no evident toxicity. Additionally, Shang et al. used therapeutic hyaluronic-acid derivatives (PAM-HA) as carriers to load sinomenine, forming controlled-release nanoparticles (PAM-HA@SIN nanoparticles) that, in high-ROS environments, undergo methionine oxidation to trigger drug release (Fig. 15D) [144]. The system effectively suppressed pro-inflammatory cytokines while upregulating anti-inflammatory mediators (Fig. 15E); in vivo, it markedly ameliorated joint inflammation and cartilage damage with an excellent safety profile (Fig. 15F).

Beyond conventional nanoparticles, stimuli-responsive hydrogel materials have also been widely applied. Wang et al. developed a sodium alginate hydrogel with dual dynamic crosslinking, incorporating borate-ester linkages and tea-polyphenol moieties; this hydrogel scavenges excess ROS on demand and provides stable, sustained release of triptolide, thereby promoting cartilage regeneration and alleviating inflammation [145]. It also exhibits self-healing and tissue adhesiveness, ensuring long-term, uniform drug delivery, with high biocompatibility. Furthermore, Tang et al. constructed neutrophil-membrane-camouflaged nanoliposomes that leverage CAT to eliminate ROS while synergistically releasing leonurine, enabling precise enrichment at inflammatory sites and regulation of macrophage repolarization [146,147]. This system efficiently scavenges ROS, downregulates HIF-1α, and, via its cell-membrane cloak, enhances targeting to inflamed tissues. Animal studies further confirmed its excellent therapeutic efficacy.

Overall, mono-stimuli-responsive nanomaterials enable efficient, low-toxicity RA therapy by precisely sensing and scavenging ROS within the arthritic microenvironment while enabling on-demand release of anti-inflammatory drugs or antioxidant cargos [148]. Ongoing innovations in delivery architecture, materials engineering, and safety optimization across diverse platforms are collectively accelerating the advancement and clinical promise of ROS-responsive nanotherapeutic systems [149,150].

#### 2.3.2 Multi-stimuli-responsive nanosystems with ROS scavenging

RA lesions are characterized not only by elevated ROS but also by complex microenvironmental features, including reduced pH, aberrant enzyme expression, and temperature fluctuations [151]. In response, multi-stimuli-responsive nanosystems have been developed that integrate synergistic triggers, such as ROS, pH, NO, and temperature, to achieve precise drug release under high-ROS conditions while leveraging pH-sensitive motifs to enhance localization at inflamed sites [[152], [153], [154]]. These platforms can also interface with physical cues (e.g., microwave irradiation) to modulate penetration depth and spatiotemporal delivery profiles [80]. By dynamically adapting to microenvironmental gradients (e.g., pH/ROS), multi-responsive systems enable coordinated regulation and synchronous intervention across multiple pathological axes, thereby overcoming the constraints of traditional single-target therapies and offering a more precise, programmable strategy for RA across diverse subtypes and disease stages [[155], [156], [157]].

Wang et al. developed a ROS/pH dual-responsive hydrogel composed of phenylboronic-acid-grafted poly-L-lysine/oxidized-dextran-selenium nanoparticles that synchronously scavenges ROS and buffers local acidity under ROS-rich, mildly acidic conditions [158,159]. This dual responsiveness alleviates oxidative stress while concurrently enhancing the hydrogel's mechanical robustness. By suppressing PI3K/AKT/NF-κB and MAPK pathways, the platform reduces TNF-α, IL-6, and IL-1β, enhances IL-10 secretion, and consequently shifts macrophages from an M1 to an M2 phenotype. In vitro, the hydrogel increased SOD and glutathione-peroxidase activities while reducing malondialdehyde levels; in vivo, it significantly mitigated joint inflammation and bone damage.

For targeting-oriented delivery, Lu et al. engineered a pH/ROS dual-responsive nanoplatform based on α-cyclodextrin to precisely deliver methylprednisolone, which suppresses TNF-α release from activated monocytes/macrophages and modulates NF-κB transcriptional activity [160]. The design employs a CA acetal linkage and 4-(hydroxymethyl) phenylboronic acid pinacol ester (HPAP) moiety that respond to low pH and elevated H_2_O_2_, respectively, enabling intelligent, site-specific release within inflamed tissue, while an RGD-peptide coating enhances accumulation in macrophages and synovial cells. Both in vitro and in CIA mice, the system efficiently scavenged ROS, downregulated NF-κB signaling and inflammatory cytokines, markedly alleviated cartilage damage and synovial inflammation, and promoted M1 to M2 macrophage repolarization. From a materials-innovation perspective, Ge et al. developed a multi-arm PEG-modified covalent organic framework photothermal composite that is dual-responsive to high ROS and low pH, enabling delivery of the protein drug ribonuclease A to inflamed joints [161,162]. When combined with PTT, the platform eradicated aberrant fibroblast-like synoviocytes (FLSs), scavenged intracellular ROS, lowered M1 markers (TNF-α, IL-6), and increased M2 markers (CD206, IL-10). In the AIA model, it improved arthritis scores and histological repair, highlighting its potential for RA therapy.

**Fig. 16:**
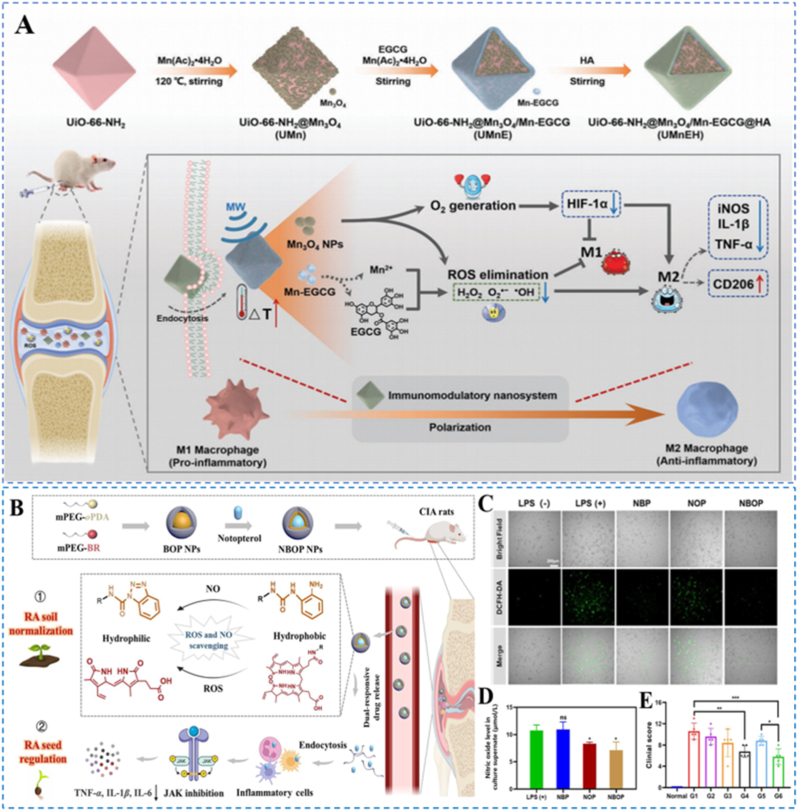
(A) Diagram depicting the fabrication workflow of UMnEH and the corresponding in vivo mechanism of action in RA. Reproduced with permission from Ref. [163]. Copyright Wiley 2023. (B) Diagram depicting the preparation of Not@BR/oPDA-PEG (NBOP) nanoparticles and their therapeutic mode of action. (C) Intracellular ROS visualized by fluorescence imaging. (D) NO level in culture supernatant. (E) Clinical scores of the rats at the end of the therapy. Reproduced with permission from Ref. [164]. Copyright Elsevier 2023.

For therapeutic synergy, Chen et al. engineered a microwave-responsive metal-organic framework (UiO-66-NH_2_) based platform whose surface was coated with Mn_3_O_4_ and whose pores were loaded with manganese-modified EGCG and HA, forming the multifunctional nanoplatform UMnEH (Fig. 16A) [163]. Under mildly acidic, ROS-rich conditions and upon microwave irradiation, the Mn-oxide nanozyme shell undergoes catalytic activation/disassembly, thereby synergistically enhancing ROS scavenging, generating O_2_ to relieve hypoxia, and improving joint lubrication via HA. In vitro and in vivo, the platform effectively eliminated H_2_O_2_ and facilitated M1 to M2 macrophage polarization. When combined with microwave thermotherapy in vivo, UMnEH significantly reduced joint erythema and swelling and facilitated cartilage repair.

Peng et al. developed ROS/NO dual-responsive nanoparticles (NBOP nanoparticles) for RA therapy that covalently conjugate BR and o-phenylenediamine (oPDA) to PEG and encapsulate notopterol, a JAK-STAT pathway inhibitor (Fig. 16B) [164]. Passive enrichment of NBOP nanoparticles at inflamed joints occurs through the ELVIS effect, combining leaky-vessel extravasation with inflammatory cell-mediated sequestration. Under elevated ROS and NO, the carriers undergo oxidative/nitrosative transformations that trigger co-release of notopterol, while BR and oPDA, both of which are redox-active, efficiently scavenge ROS and NO (Fig. 16C–D). Following cellular internalization, notopterol suppresses JAK signaling and reduces pro-inflammatory cytokine expression. In vitro, NBOP nanoparticles markedly lowered intracellular ROS and NO levels. In CIA mice, they significantly alleviated arthritis symptoms and clinical scores (Fig. 16E), produced the mildest synovial inflammation and tissue damage, and shifted macrophages from an M1 toward an M2 phenotype. This dual-responsive platform couples ROS/NO scavenging with JAK pathway modulation, offering a precise strategy for RA treatment.

Overall, multimodal ROS-responsive scavenging nanosystems integrate dynamic chemistries sensitive to ROS, pH, and temperature. The components, cell models, and animal models are summarized in Table 2. This enables targeted release of therapeutic or antioxidant agents, significantly reduces ROS levels at inflammatory sites, and allows synergistic multi-drug therapy. The system effectively blocks pro-inflammatory signaling while maintaining a favorable biosafety profile, thus representing a promising strategy for targeted and spatiotemporally controlled treatment of RA.

**Table 2:** ROS-responsive and scavenging composite nanosystems: scavenging agents, stimulus triggers, and RA models.

Scavenging Agents	Stimuli Triggers	Cell Models	Animal Models	Ref.
Oxi-αCD	Oxi-αCD	RAW 264.7 cells; MH7A cells	CIA DBA/1 mice	[132]
di-S-PC	di-S-PC	RAW 264.7 cells	AIA SD rats	[135]
Ceria oxide	Thioketal linker	RAW 264.7 cells	CIA Wistar rats	[142]
thioether	thioether	RAW 264.7 cells	CIA DBA/1 mice	[143]
Polyphenol	Boronic esters	Bone marrow mesenchymal stem cells; RAW 264.7 cells; ATDC5 cells	CIA SD rats	[145]
CAT	CAT	RAW 264.7 cells; MH7A cells	AIA SD rats	[146]
Se NPs	Phenylboronic acid	RAW 264.7 cells; L929 cells	CIA SD rats	[159]
Methylprednisolone	HPAP	RAW 264.7 cells; MH7A cells	CIA DBA/1 mice	[160]
Mn_3_O_4_	EGCG	RAW 264.7 cells	AIA SD rats	[163]
BR	BR and oPDA	RAW 264.7 cells	CIA Wistar rats	[164]

### 2.4 ROS-augmented nanosystems for RA treatment

**Fig. 17:**
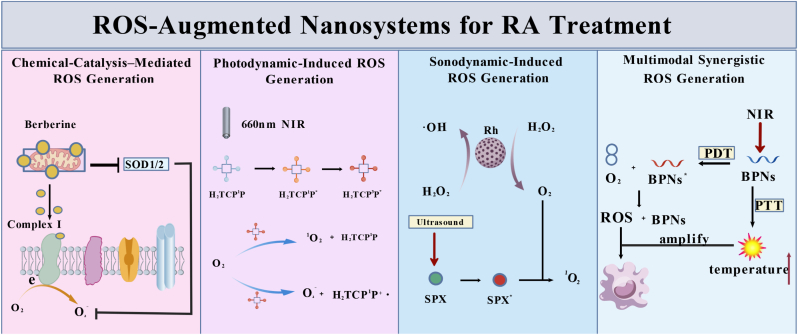
Schematic illustration of the ROS-augmented mechanism of nanosystems in RA treatment.

In the RA synovial microenvironment, aberrant proliferation of FLSs and sustained activation of M1 macrophages are central drivers of persistent inflammation, joint destruction, and disease progression [165,166]. A controlled augmentation of ROS can surmount the antioxidant defenses of FLSs and M1 macrophages, trigger mitochondrial caspase-dependent apoptosis (e.g., cytochrome *c* release and caspase-9/3 activation), and thereby suppress inflammatory cascades, cartilage degradation, and pathological synovial overgrowth [[20], [21], [22], [23], [24], [25], [26], [27], [28], [29], [30], [31], [32], [33], [34], [35], [36], [37], [38], [39], [40], [41], [42], [43], [44], [45], [46], [47], [48], [49], [50], [51], [52], [53], [54], [55], [56], [57], [58], [59], [60], [61], [62], [63], [64], [65], [66], [67], [68], [69], [70], [71], [72], [73], [74], [75], [76], [77], [78], [79], [80], [81], [82], [83], [84], [85], [86], [87], [88], [89], [90], [91], [92], [93], [94], [95], [96], [97], [98], [99], [100], [101], [102], [103], [104], [105], [106], [107], [108], [109], [110], [111], [112], [113], [114], [115], [116], [117], [118], [119], [120], [121], [122], [123], [124], [125], [126], [127], [128], [129], [130], [131], [132], [133], [134], [135], [136], [137], [138], [139], [140], [141], [142], [143], [144], [145], [146], [147], [148], [149], [150], [151], [152], [153], [154], [155], [156], [157], [158], [159], [160], [161], [162], [163], [164], [165], [166], [167]]. When coupled with O_2_-supplying or hypoxia-modulating modules, this approach can also alleviate local hypoxia and help rebalance immune homeostasis. We refer to this class of interventions where selective cellular ablation is achieved via on-site ROS elevation as “ROS-augmented RA nano-therapy”, encompassing chemical catalysis, PDT, sonodynamic therapy (SDT), and multimodal synergistic platforms that confine ROS generation spatiotemporally to diseased tissues (Fig. 17). Accumulating evidence indicates that these nanotechnologies enable precise elimination of key pro-inflammatory cell populations and offer a promising pathway toward efficient, selective, and precise RA treatment [168].

#### 2.4.1 Chemical-catalysis–mediated ROS generation

**Fig. 18:**
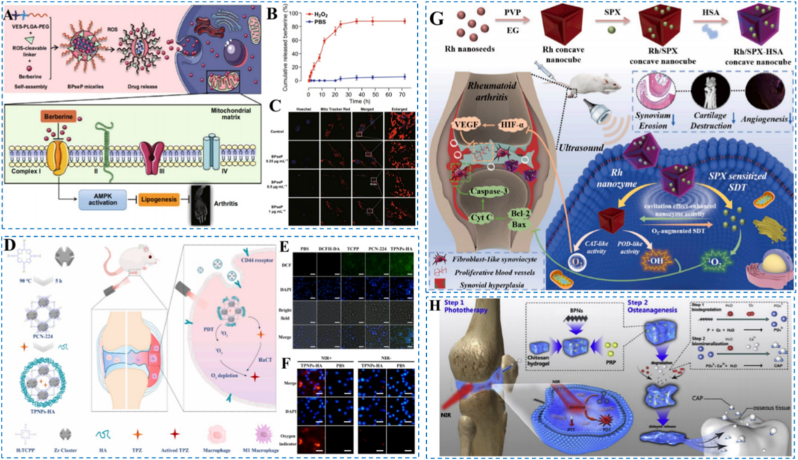
(A) Preparation workflow of BPseP with a schematic overview of its mechanisms for treating RA. (B) The release of berberine in BPseP micelles. (C) The morphology changes of mitochondria. Reproduced with permission from Ref. [169]. Copyright Springer 2020. (D) Schematic depiction of TPNPs-HA fabrication and the mechanism enabling selective targeting of activated macrophages for synergistic RA control. (E) Fluorescence microscopy readouts of intracellular ROS. (F) Fluorescence microscopy readouts of intracellular O_2_. Reproduced with permission from Ref. [174]. Copyright Elsevier 2024. (G) Diagram depicting the fabrication of Rh-SPX/HSA and its therapeutic mechanisms against RA. Reproduced with permission from Ref. [178] Copyright Elsevier 2021. (H) Overview schematic of the PRP-chitosan thermo-responsive hydrogel in combination with black phosphorus (BP) nanosheets for RA biotherapy and phototherapy. Reproduced with permission from Ref. [38]. Copyright Elsevier 2020.

Chemical catalysis mediated ROS generation refers to the use of specific small molecules or catalysts that participate in cellular or subcellular redox reactions to promote ROS formation. For example, berberine perturbs the mitochondrial electron-transport chain, directly engaging redox processes and increasing electron leak to O_2_ to yield ROS; it can also interact with intracellular proteins or receptors to initiate signaling cascades that further impair mitochondrial function and elevate ROS production [167]. Such strategies provide a mechanistic basis and delivery direction for RA therapy by tuning endogenous ROS levels. Building on this concept, Fan et al. assembled a multifunctional nanoplatform from VPseP copolymers and berberine for targeted anti-inflammatory therapy in RA (Fig. 18A) [169]. At inflamed sites, the nano-micelles disassemble under high-ROS stimulation to precisely release berberine (Fig. 18B). Once internalized, berberine targets mitochondria, increases superoxide, and activates AMPK, thereby suppressing lipid synthesis and cell proliferation to exert anti-RA effects. Experiments demonstrated that the nano-micelles enhanced cellular uptake, and immunoblotting/fluorescence assays confirmed elevated mitochondrial superoxide, G2-phase arrest of FLSs, and inhibition of inflammatory progression (Fig. 18C). In vivo, the low-dose nanomicelle group outperformed a higher-dose free-drug group, underscoring superior delivery efficiency and anti-inflammatory efficacy.

#### 2.4.2 Photodynamic therapy (PDT)-Induced ROS generation

Light-induced ROS generation employs photosensitizers that, upon irradiation at defined wavelengths, transfer energy or electrons to molecular oxygen to produce ROS, thereby inducing cytotoxicity or functional modulation [170]. Conventional PDT for RA has been limited by suboptimal ROS yield and lesion selectivity, whereas nanotechnology markedly improves precision and efficacy [[171], [172], [173]]. Accordingly, Zhang et al. designed a hyaluronic-acid-decorated, light-responsive targeted system (TPNPs-HA) that efficiently delivers the hypoxia-activated prodrug tirapazamine (TPZ) to activated M1 macrophages (Fig. 18D) [174,175]. Within TPNPs-HA, a porphyrinic metal-organic framework generates abundant ROS under near-infrared irradiation (Fig. 18E and F), while hypoxia in inflamed joints activates TPZ, yielding PDT-hypoxia-chemotherapy synergy that selectively eliminates pathogenic M1 macrophages and suppresses inflammatory cascades [176]. In vivo, this platform effectively mitigated RA pathology, underscoring its potential for targeted, synergistic therapy.

#### 2.4.3 Sonodynamic therapy (SDT)-Induced ROS generation

Sound-driven therapy employs ultrasound-responsive sensitizers to generate ROS via energy or electron transfer to molecular oxygen, thereby modulating the inflammatory microenvironment and suppressing inflammatory-cell activation to alleviate RA inflammation [177]. Li and colleagues developed a concave, cube-shaped rhodium nanozyme incorporating the sonosensitizer sparfloxacin (SPX) together with human serum albumin (Fig. 18G) [178]. Within inflamed joints, ultrasound triggers SPX release from the nanozyme, while the rhodium catalyst exhibits catalase and peroxidase-like (CAT/POD-like) activities. CAT decomposes H_2_O_2_ to O_2_ and H_2_O, whereas POD catalyzes H2O2-dependent oxidation to generate ·OH, thereby relieving hypoxia and inhibiting angiogenesis. Ultrasound further drives SPX to generate ^1^O_2_, and together with ·OH produced by the nanozyme, these species activate the mitochondrial caspase cascade to induce apoptosis of FLSs, suppressing synovial proliferation and cartilage destruction. Acoustic cavitation enhances nanozyme activity and accelerates ROS generation, while nanozyme-derived O_2_ sustains SPX-mediated 1O_2_ production, yielding synergistic therapeutic effects. Cellular and animal studies demonstrated efficient induction of FLS apoptosis, attenuation of inflammatory responses, and superior anti-RA efficacy.

#### 2.4.4 Multimodal synergistic ROS generation (PTT/SDT/PDT)

The photothermal effect refers to the conversion of light into localized heat by photothermal materials, which elevates the peri-lesional temperature, facilitates ROS generation, and induces apoptosis of pathogenic inflammatory cells [179,180]. Photoacoustic-/sonodynamic-based approaches employ acoustic sensitizers that are activated by pulsed light or ultrasound; via thermoelastic expansion and acoustic cavitation, these sensitizers interact with O_2_ to efficiently generate ROS [181]. When combined, photothermal heating augments acoustic/chemical ROS yield and improves lesion selectivity, producing a more potent and spatially confined ROS assault on hyperplastic synovium and other pathogenic cells. Relative to either modality alone, PTT/SDT synergy not only enhances ROS production and inflammatory-site targeting but also promotes bone and cartilage repair and improves joint function, offering a precise and efficient strategy for RA therapy.

Pan et al. engineered a multifunctional hydrogel incorporating black phosphorus (BP) nanosheets to realize synergistic PTT/PDT for RA (Fig. 18H) [38]. Under near-infrared irradiation, BP nanosheets concurrently generate localized hyperthermia and ROS-hyperthermia suppresses hyperplastic synovium, while ROS induce apoptosis to eliminate aberrant inflammatory cells. Biodegradation of BP yields phosphate species that promote biomineralization and bone regeneration, and the incorporation of platelet-rich plasma and a chitosan hydrogel matrix enhances stem-cell adhesion/proliferation and augments cartilage protection. Collectively, the platform couples high photothermal-conversion efficiency with robust ROS generation, biocompatibility, and in vivo reparative effects.

Tang et al. introduced a photodynamic-sonodynamic synergistic strategy by engineering poly (lactic-co-glycolic acid) (PLGA) phase-change nanoparticles co-loaded with O_2_ and ICG [182]. Upon near-infrared irradiation, the particles undergo a phase transition that releases microbubbles; these microbubbles activate ICG to generate abundant ROS via PDT and trigger apoptosis. Subsequent low-intensity ultrasound ruptures the microbubbles, further liberating O_2_ and ICG to potentiate (SDT), thereby achieving stronger, synergistic ROS production. Experiments confirmed high ROS output, efficient cellular uptake, and favorable biodegradability and safety conferred by the PLGA matrix; the combination group yielded the highest apoptosis rates, validating the PDT/SDT synergy. In a complementary approach, Jin et al. devised a gelatinase matrix metalloproteinase-9 (MMP-9) responsive smart nanotheranostic platform that integrates chemotherapy, radiosensitization, and near-infrared imaging [183]. Gelatin-coated cisplatin (DDP) and ICG undergo shell degradation within MMP-9-rich inflamed joints, enabling precise drug release; DDP enhances ROS generation in concert with X-ray radiotherapy, while ICG provides both photothermal effects and NIR-II imaging [184]. In vitro and in vivo studies demonstrated efficient lesion enrichment, elevated intra-articular ROS, increased synovial-cell apoptosis, mitigation of bone erosion and synovial hyperplasia, and an excellent safety profile showcasing an “enzyme-responsive-image-guided-multimodal sensitization” paradigm for precise RA therapy applications.

Overall, multimodal synergistic ROS-generating nanoplatforms integrate physical (light, sound, heat), chemical, and biological triggers to markedly enhance ROS yield and lesion selectivity, while enabling synergistic imaging therapy, promoting tissue repair, and maintaining favorable safety profiles together offering strong potential for coordinated, multi-target regulation of RA-associated inflammation and osteoarticular damage. The components, cell models, and animal models of the ROS-enhanced nanosystems are summarized in Table 3.

**Table 3:** ROS-augmented nanosystems: agents and RA models.

Agents	Cell Models	Animal Models	Ref.
Berberine	HFLS cells	AIA SD rats	[169]
TPZ	RAW 264.7 cells	AIA SD rats	[174]
Rhodium nanoenzyme	FLS cells	CIA rats	[178]
BP	RAW 264.7 cells	CIA DBA/1 mice	[38]
ICG	MH7A cells	None	[182]
DDP	RASF cells	CIA DBA/1mice	[183]

## 3 Prospects and outlook

In the pathogenesis of RA, ROS occupy a central role in driving synovial inflammation and joint damage, and nanomaterial-based ROS-regulation strategies are reshaping therapeutic paradigms. First, nanosystems can sense the high-ROS microenvironment at lesional sites to enable precise, on-demand drug release, thereby improving local bioavailability while limiting systemic adverse effects; for instance, architected carriers that undergo ROS-triggered disassembly can preferentially release payloads within inflamed tissues to enhance efficacy [185]. Second, many nanomaterials possess intrinsic antioxidant activity, enabling efficient scavenging of excess ROS, alleviation of intra-articular oxidative stress, and protection of synovial and cartilage tissues, which together contribute to disease remission and functional recovery [64]. For example, some metal-based ROS-modulating systems display high attraction because they can catalytically decompose large amounts of ROS and, in some cases, simultaneously generate O_2_ to relieve intra-articular hypoxia, while also offering intrinsic imaging and theranostic potential. Third, the multifunctionality of nanodrug platforms supports comprehensive treatment: they can co-deliver anti-inflammatory and immunomodulatory agents and be combined with photodynamic or sonodynamic modalities to produce local ROS bursts that potentiate therapeutic effects [186]. It is important to note that the frequent incorporation of ROS-scavenging modules together with anti-inflammatory or disease-modifying agents in many platforms does not indicate that ROS regulation is intrinsically ineffective. Rather, it reflects the multifactorial nature of RA, in which oxidative stress, cytokine networks, synovial hyperplasia, and osteoclastogenesis form tightly coupled feed-forward loops. In this context, ROS-targeted components act as key “circuit breakers” that disrupt redox-driven amplification, while co-delivered anti-inflammatory or immunomodulatory drugs address complementary pathogenic axes, thereby achieving more durable disease control. Concurrently, nanomaterials show promise for early diagnosis and inflammation imaging, providing technical support for precision diagnostics and stratified therapy in RA [187,188].

Despite substantial progress, the clinical translation of nanomaterials still faces several hurdles. The foremost challenge is in vivo stability and biocompatibility: nanoparticles may aggregate, degrade prematurely, or elicit foreign-body responses, thereby compromising targeting and durable therapeutic efficacy. Systematic elucidation of biodistribution, metabolic clearance pathways, and long-term safety profiles of nanosystems remains a critical technical bottleneck to overcome [189,190]. For example, for metal-based nanozymes such as ceria, preclinical studies have shown predominant accumulation in liver and spleen through uptake by the mononuclear phagocyte system, followed by slow hepatobiliary or renal elimination that depends on particle size, surface chemistry, and dosing regimen [191]. These biodistribution and clearance patterns highlight the need for long-term follow-up, careful dose optimization, and rational surface engineering to minimize potential in vivo toxicity while maintaining therapeutic benefit. A second challenge is the need for precise control of ROS generation and scavenging during therapy excess ROS exacerbate cellular injury, whereas insufficient levels may fail to engage immune-clearance mechanisms; individualized, spatiotemporally dynamic regulation of ROS homeostasis is therefore essential for efficacy with minimal toxicity [192,193]. Third, bench-to-bedside translation is impeded by process standardization, manufacturing controllability, batch-to-batch consistency, and rigorous validation of safety and efficacy; achieving scalable, controllable GMP production and navigating regulatory review will be pivotal for industrialization [194]. Finally, the lack of real-time, clinically deployable ROS quantification limits response assessment and adaptive therapy; the development of bedside-ready diagnostic tools capable of longitudinal in vivo ROS monitoring is urgently needed to support precise evaluation and dynamic adjustment of nanotherapeutic regimens [195].

From a therapeutic standpoint, ROS-modulating nanomedicines should be viewed as adjuncts to, rather than replacements for existing RA therapies (e.g., TNF-α and JAK inhibitors). By intervening at the level of synovial oxidative stress, hypoxia, and redox-sensitive signaling pathways, ROS-oriented nanoplatforms can theoretically enhance responses to standard agents, reduce the required doses, and mitigate off-target toxicity, particularly in patients with partial responses or contraindications to aggressive immunosuppression. In this sense, programmable ROS modulation is best positioned as a complementary pillar within multi-target regimens that integrate immune, cytokine, and redox control. From a translational perspective, nanoplatforms constructed from FDA-approved carriers, such as liposomes, offer clear advantages in terms of safety profiles, scalable GMP-compliant manufacturing, and regulatory familiarity. In contrast, nanomaterials that have not yet been clinically validated, such as cerium oxide, require more cautious assessment of their long-term biodistribution, potential tissue accumulation and clearance pathways, as well as more stringent quality control and batch-to-batch reproducibility to meet regulatory standards. In addition, systematic evaluation of the immunogenicity and pharmacokinetics of these nanocarriers is crucial for advancing ROS-modulating nanotherapeutic strategies from experimental RA models toward clinical application.

## 4 Conclusion

In summary, ROS play a central regulatory role in the pathogenesis and progression of RA by activating inflammatory signaling pathways and influencing immune cell polarization as well as redox homeostasis. Based on this pathophysiological feature, the construction of ROS-modulating nanotherapeutic systems provides new strategies for precise RA intervention. Current studies have demonstrated that various nanoplatforms, including ROS-scavenging, ROS-responsive, composite, and ROS-augmenting systems, exhibit remarkable advantages in remodeling the inflammatory microenvironment, preventing cartilage destruction, and promoting tissue repair. Compared with conventional pharmacotherapies, these programmable nanoplatforms possess superior targeting capability, improved drug utilization, and reduced systemic toxicity, and they can further integrate drug delivery with imaging monitoring through multimodal design. Nevertheless, the clinical translation of ROS-regulating nanosystems still faces several challenges, such as long-term biosafety, in vivo metabolic pathways, and manufacturing controllability. Future research should focus on developing more intelligent, biocompatible, and biodegradable nanoplatforms, integrating multi-omics analysis and advanced imaging techniques to achieve real-time monitoring of ROS dynamics and personalized intervention throughout RA progression. Overall, ROS-modulating nanotherapeutic strategies provide a new theoretical foundation and developmental direction for the precise diagnosis and treatment of RA, holding great promise for bridging experimental research and clinical application.

## CRediT authorship contribution statement

**Guojun Pan:** Writing – original draft, Methodology, Funding acquisition. **Yaxin Zhang:** Writing – original draft, Data curation. **Di Liu:** Visualization, Data curation. **Yongbin Wang:** Formal analysis, Conceptualization. **Hengzhen Zhang:** Data curation. **Haoen Pan:** Writing – review & editing. **Qingfan Hou:** Formal analysis. **Xiangrui Kong:** Visualization. **Fujin Lv:** Conceptualization. **Na Xiao:** Writing – review & editing, Investigation. **Renshuai Zhang:** Writing – review & editing, Project administration, Funding acquisition.

## Funding

This work was supported by grants from the 10.13039/501100007129Natural Science Foundation of Shandong Province, China (ZR2021MB087, ZR2021QH212).

## Declaration of competing interest

The authors declare that they have no known competing financial interests or personal relationships that could have appeared to influence the work reported in this paper.

## Data Availability

No data was used for the research described in the article.
